# Quasi-phase-matching-division multiplexing holography in a three-dimensional nonlinear photonic crystal

**DOI:** 10.1038/s41377-021-00588-5

**Published:** 2021-07-15

**Authors:** Pengcheng Chen, Chaowei Wang, Dunzhao Wei, Yanlei Hu, Xiaoyi Xu, Jiawen Li, Dong Wu, Jianan Ma, Shengyun Ji, Leran Zhang, Liqun Xu, Tianxin Wang, Chuan Xu, Jiaru Chu, Shining Zhu, Min Xiao, Yong Zhang

**Affiliations:** 1 National Laboratory of Solid State Microstructures, College of Engineering and Applied Sciences, School of Physics, and Collaborative Innovation Center of Advanced Microstructures, Nanjing University, Nanjing, 210093 China; 2 Hefei National Laboratory for Physical Sciences at the Microscale and CAS Key Laboratory of Mechanical Behavior and Design of Materials, Department of Precision Machinery and Precision Instrumentation, University of Science and Technology of China, Hefei, 230026 China; 3 Department of Physics, University of Arkansas, Fayetteville, AR 72701 USA

**Keywords:** Nonlinear optics, Laser material processing

## Abstract

Nonlinear holography has recently emerged as a novel tool to reconstruct the encoded information at a new wavelength, which has important applications in optical display and optical encryption. However, this scheme still struggles with low conversion efficiency and ineffective multiplexing. In this work, we demonstrate a quasi-phase-matching (QPM) -division multiplexing holography in a three-dimensional (3D) nonlinear photonic crystal (NPC). 3D NPC works as a nonlinear hologram, in which multiple images are distributed into different Ewald spheres in reciprocal space. The reciprocal vectors locating in a given Ewald sphere are capable of fulfilling the complete QPM conditions for the high-efficiency reconstruction of the target image at the second-harmonic (SH) wave. One can easily switch the reconstructed SH images by changing the QPM condition. The multiplexing capacity is scalable with the period number of 3D NPC. Our work provides a promising strategy to achieve highly efficient nonlinear multiplexing holography for high-security and high-density storage of optical information.

## Introduction

Quasi-phase-matching (QPM) theory has boosted the development of nonlinear optics for decades because it can significantly enhance the conversion efficiency of nonlinear optical processes^1^. The principle of QPM is to utilize a periodically modulated *χ*^(2)^ [which is equivalent to a reciprocal vector (RV)] to compensate for the phase mismatch between the interacting waves. Artificially-engineered nonlinear photonic crystal (NPC)^2^ is one of the most popular materials to realize QPM, which has been widely investigated in laser frequency conversion^3,4^, nonlinear optical imaging^5^, and quantum light sources^6,7^. Typical one-dimensional (1D) and two-dimensional (2D) NPCs include periodically-poled LiNbO_3_ crystals by applying electric fields^8,9^ and metasurface NPCs through micro/nano-fabrications^10,11^. Recently, three-dimensional (3D) NPCs were successfully realized by employing femtosecond laser writing techniques^12,13^, which provide a powerful platform to manipulate nonlinear waves under 3D configuration for unprecedented applications^14,15^.

Holography is capable to reconstruct both the intensity and phase information of an object, which has been widely applied in optical display^16,17^, data storage^18,19^, information security^20^, and microscopy^21^. In recent years, the concept of holography has been extended to nonlinear optics, leading to new domains in nonlinear holography^22–30^. Since the encoded information is reconstructed in the newly-generated wavelength, nonlinear holography has been viewed as a promising technique for high-density optical storage and high-security optical encryption. 2D NPC is the popular platform to realize nonlinear holography^24,30,31^. However, it can only provide 2D modulation of nonlinear interacting waves, which severely limits the performance of nonlinear holography. For example, the general configuration in previous works is typically based on nonlinear Raman-Nath diffraction^32^, in which the phase-matching condition is partially satisfied (i.e., there still exists a certain residual phase mismatch). Therefore, the typical conversion efficiency of the reported nonlinear holography is 10^-6^ or less^33^. Another key issue in demonstrating nonlinear holography is its ineffective multiplexing/demultiplexing capability. Only a few schemes have been reported that can achieve nonlinear multiplexing holography with limited channel numbers^15,22,23,34^. For example, in a specially designed metasurface, spin angular momentum is introduced to encode two different patterns at the second-harmonic (SH) wave^22^. However, the multiplexing capacity, as well as the conversion efficiency, need to be significantly enhanced for practical applications.

**Fig. 1:**
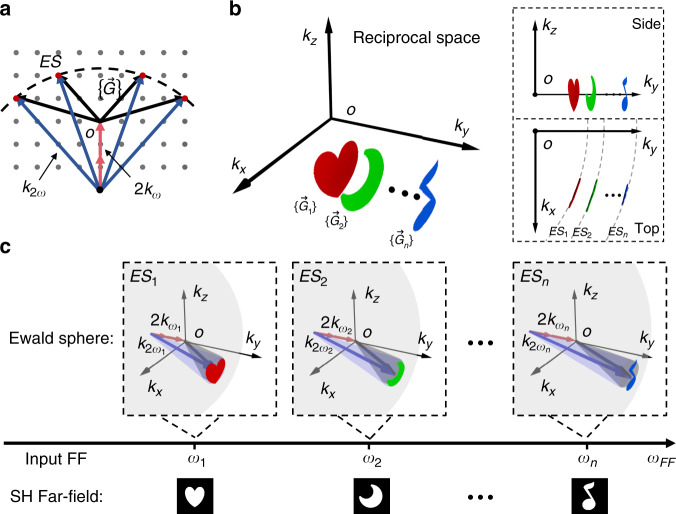
Illuminating QPM-division multiplexing nonlinear holography through nonlinear Ewald construction in reciprocal space. The center of the Ewald sphere (*ES*) is located 2*k*_*ω*_ away from the origin (*O*) of the reciprocal lattice and the radius of the sphere is *k*_2*ω*_. Here, *k*_*ω*_ and *k*_2*ω*_ are the wave vectors of the fundamental wave and SH wave, respectively. **a** shows a traditional periodic reciprocal lattice, in which several discrete points (red) locating on the Ewald sphere (dashed line) provide RVs ($\{ {\vec G} \}$) to participate in the QPM process. **b** gives aperiodic distribution of RVs for QPM-division multiplexing holography. The red, green, and blue patterns represent various groups of RVs on different Ewald spheres (*ES*_*1*_, *ES*_*2*_, … *ES*_*n*_). **c** is the schematic of QPM-division multiplexing nonlinear holography. When using different fundamental frequencies (FF) *(ω*_1_, *ω*_2_, ……, *ω*_n_), one can tune the Ewald sphere to pick out the designed RV group and generate distinctive SH far-field images

In this Letter, we report a QPM-division multiplexing nonlinear holography in a 3D NPC. The principle can be understood through considering nonlinear Ewald construction^2^ in reciprocal space (Fig. 1a). If reciprocal lattice points are located on the Ewald sphere, they will provide a collection of RVs (i.e.,$\{ {\vec G} \}$) to satisfy the complete QPM condition for the SH generation process (Fig. 1a). Correspondingly, the generated SH wave carries the spatial frequency information of this group of $\{ {\vec G} \}$. Different from traditional periodic reciprocal lattice (Fig. 1a), nonlinear holography based on nonlinear Ewald construction requires that RVs are extended in 3D reciprocal space to form a designed distribution on the Ewald sphere (Fig. 1b). Notably, only 3D NPC is capable to efficiently accomplish this task under complete QPM configuration (see Supplementary Note 1 for the comparison to 2D NPC). Ewald sphere can be feasibly tuned by changing the input wavelength, which will select a different group of RVs (Fig. 1c). When encoding multiple images into various Ewald spheres, one can selectively reconstruct them at SH wave by satisfying the corresponding QPM condition, i.e., QPM-division multiplexing holography (Fig. 1c). The minimal wavelength-division is fundamentally decided by QPM bandwidth (typically several nanometers). The proposed technique can well promote the application of nonlinear holography for high-density optical storage and high-security optical encryption.

## Results

### The theory of QPM-division multiplexing nonlinear holography

The *z*-polarized fundamental beam propagates along the *y*-axis of 3D NPC. The distribution of $\chi ^{(2)}$ is written as $\chi ^{\left( 2 \right)}\left( {\overrightarrow {\mathrm{r}} } \right) = 2d_{33} \times f\left( {\vec r} \right)$ with $f\left( {\vec r} \right)$ being the structure function. Under slowly-varying envelope approximation, the nonlinear wave-mixing equation for SH generation can be written as^35^

1 $$\vec k_{2\omega } \cdot \nabla E_{2\omega }\left( {\vec r} \right) = - i\frac{{4\omega ^2}}{{c^2}}d_{33}f\left( {\vec r} \right)E_\omega \left( {\vec r} \right)E_\omega \left( {\vec r} \right)e^{i\left( {\vec k_{2\omega } - 2\vec k_\omega } \right) \cdot \vec r}$$

where $E_\omega$ and ${\mathrm{E}}_{2\omega }$ are the electric fields of fundamental and SH waves, respectively. The QPM condition is completely fulfilled in such 3D NPC, i.e., $\vec k_{2\omega } - 2\vec k_\omega - \vec G = 0$. From Eq. (1), the far-field SH wave satisfies^36^

2 $$E_{2\omega ,far} \propto {\it{F}}( {\{ {\vec G} \}} )$$

here, *F* is the Fourier coefficient of RVs, which is calculated by Fourier transform of ${\it{f}}\left( {\vec r} \right)$. Equation (2) indicates that SH far-field is the mapping of the RV distribution on the designated Ewald sphere.

### Design of 3D NPC for nonlinear multiplexing holography

**Fig. 2:**
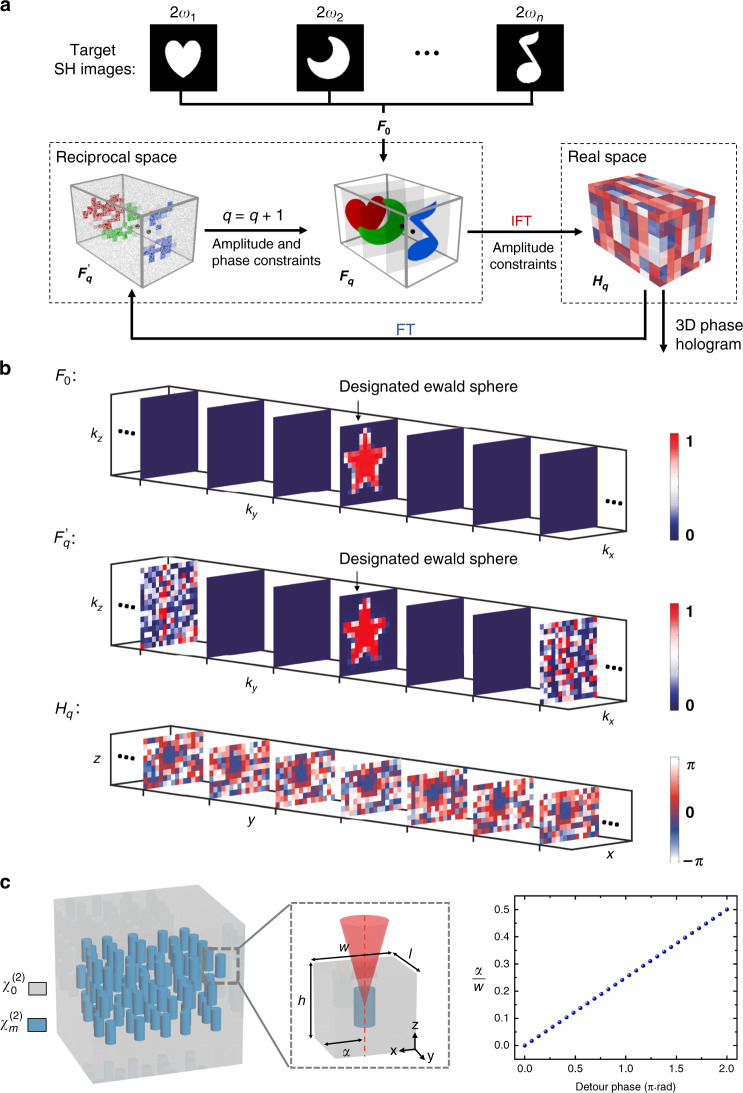
Design of 3D NPC structure. Based on 3D QPM theory, one can calculate the ideal RV distribution *F*_0_, which, however, can hardly be realized in experiments. Our strategy is to search for a 3D phase hologram *H* in real space that has an RV distribution close to *F*_0_. The schematic flow in **a** shows the procedure to use a 3D iterative Fourier transform algorithm to calculate the 3D phase hologram. We repeat the loop by *q* iterations to achieve an optimized $F_q^\prime$ and *H*_*q*_. **b** shows an example. In the ideal *F*_0_ (top row), all RVs locate on the designated Ewald sphere while there are no RVs in all other Ewald spheres. In the calculated *H*_*q*_ (bottom row), $F_q^\prime$ is almost the same as *F*_0_ in the designated Ewald sphere and its neighboring spheres (middle row). The other Ewald spheres carrying certain RVs are far away from the designated one, which have negligible influences because of phase mismatch. **c** shows the 3D NPC structure to realize *H*_*q*_ by detour phase coding. 3D NPC is divided into a 3D array of cuboid unit cells. The size of the unit cell is *w* (*x*) × *l* (*y*) × *h* (*z*). There is a laser-erased pillar in each unit and the phase of each unit is controlled by the relative position *α* of the pillar. A detour phase range of 2*π* is realized by tuning the value of *α/w* from 0 to 0.5 (see Supplementary Notes2 and 3 for detail algorithms)

Figure 2 shows how to design a 3D NPC for QPM-division multiplexing holography. According to the target images at their respective wavelengths, multiple RV groups (locating on different Ewald spheres) are calculated through 3D QPM conditions, which are combined together to form an ideal 3D distribution of RVs (*F*_0_). Considering the binary-amplitude modulation of *χ*^(2)^ and point-by-point fabrication in femtosecond laser erasing technique (see Materials and methods for detail), it is impossible to perfectly realize *F*_0_. However, one can design an NPC structure that well approaches *F*_0_. The procedure is divided into two steps. First, we use a 3D iterative Fourier transform algorithm^37,38^ (Fig. 2a) to transform the ideal *F*_0_ in reciprocal space to a practical 3D phase hologram *H* in real space. By setting the proper amplitude and phase constraints and repeating the loops in Fig. 2a by *q* iterations, one can obtain an optimized 3D phase hologram *H*_*q*_. Figure 2b shows an example of designing a 3D phase hologram. In comparison to the ideal *F*_0_, the 3D phase hologram *H*_*q*_ guarantees that the RV distribution in the designated Ewald sphere is almost the same as that in *F*_0_ while there are no RVs in the neighboring Ewald spheres. The unwanted RVs are pushed far away from the designated Ewald sphere, which have negligible effects on nonlinear holography because of substantial phase mismatch. The second step is to realize 3D phase hologram *H*_*q*_ by using detour phase coding^39^, in which the phase of each unit is controlled by the relative position of the laser-erased area (Fig. 2c).

### Experimental demonstration of QPM-division multiplexing holography in 3D NPC

**Fig. 3:**
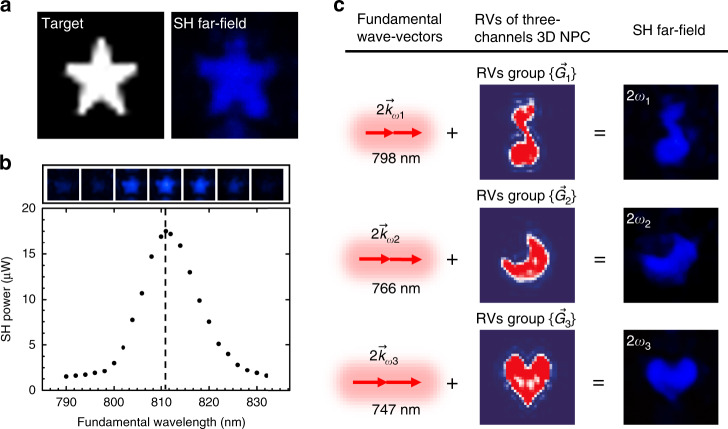
Experimental results. **a** shows the reconstruction of a star pattern at SH wave. At the QPM wavelength of 811 nm, the experimental SH pattern (right) agrees well with the target (left). **b** gives the output power and pattern at SH wave when tuning the fundamental wavelength. As predicted in theory, the SH holographic image is clear and bright at 811 nm and becomes weak and indistinguishable when the wavelength is tuned away. **c** demonstrates the experimental results of three-channel QPM-division multiplexing holography. A musical note, a moon, and a heart are encoded into three RV groups at different Ewald spheres, which are well reconstructed by using the fundamental wavelengths of 798, 766, and 747 nm, respectively

We use a femtosecond-laser-erasing technique to fabricate the 3D LiNbO_3_ NPC carrying the designed 3D hologram of 16 (*x*) × 16 (*y*) × 10 (*z*) pixels (see Materials and methods for detail). We first produce a hologram to generate a star pattern in SH wave. The target and experimental images in Fig. 3a are well consistent with each other. Figure 3b presents the dependence of the output on the fundamental wavelength. Since the 3D QPM condition is fully satisfied at an input wavelength of 811 nm, the corresponding SH pattern is well recognized with its power being highest. At an input power of 2.4 W, the conversion efficiency reaches 1.7 × 10^−5^, which is at least one order of magnitude higher than the previous reports. When the fundamental wavelength is tuned away from the QPM wavelength, the SH power drops rapidly and the SH pattern becomes indistinguishable. Figure 3c shows QPM-division multiplexing nonlinear holography. Three patterns (a musical note, a moon, and a heart) are encoded into different Ewald spheres from a single 3D NPC. When the fundamental waves interact with their respective RV groups, various QPM conditions are triggered and different SH images are reconstructed separately. See Supplementary Figs. S3 and S4 and Supplementary Table S1 for hologram and NPC structures.

**Fig. 4:**
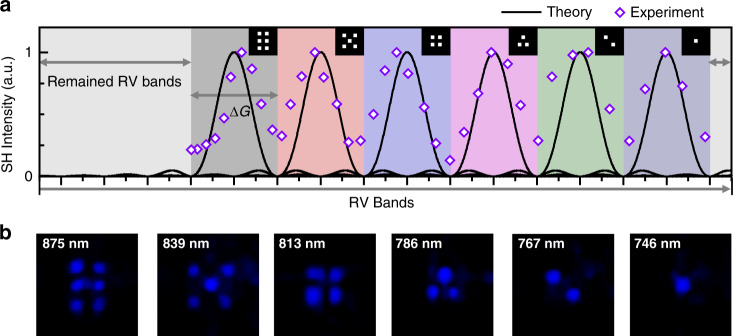
Experimental demonstration of high-density multiplexing. Under our experimental setup, the period number along the propagation direction (*y*) is *N* = 16 and the theoretical capacity can reach *N*/2 = 8. In our design, we choose a capacity of 6 for high-quality image reconstruction. **a** shows the usage of RV source along *the y* direction. The RV bandwidth for each image is set to be ${\Delta}{\mathrm{G}}$= $\frac{{4\pi }}{{{\mathrm{Nl}}}}$. When tuning the input fundamental wavelengths, different RV bands are selected to satisfy the corresponding QPM conditions. The measured QPM peaks are well consistent with the theoretical ones. The remained RV bands (light gray areas in **a**) are utilized to enhance the overall image quality. **b** shows the experimental results, i.e., six facets of dice, by using fundamental wavelengths of 875, 839, 813, 786, 767, and 746 nm, respectively. The clear background indicates that the interactions between different channels can be ignored

The capacity of QPM-division multiplexing holography mainly depends on the period number (*N*) of the unit cells along the propagation direction (*y*) (see Supplementary Note 4 for detailed calculations). In theory, the total RV bandwidth along *y-direction* is $\frac{{2\pi }}{l}$. Here, *l* is the length of the unit cell (Fig. 2c). To effectively avoid crosstalk between different channels, we define the limit as that two neighboring QPM peaks overlap at their first zero-value points, which requires an RV bandwidth $\frac{{4\pi }}{{Nl}}$ for each image (Fig. 4a). Therefore, the maximal channel number is $\frac{N}{2}$. However, in such extreme cases, the image quality (including intensity and contrast) of each channel will become too low to be well distinguished. In actual design, the multiplexing capacity is chosen to be a little less than the theoretical limit. Then, one can use the saved RV bandwidth (Fig. 4a) to improve the image quality through a 3D iterative Fourier transform algorithm (See Supplementary Note 2 for details). In our experiment with *N* = 16, we can achieve a maximal capacity of 6 with clear patterns (Fig. 4b). Notably, the wavelength interval gradually increases along with the wavelength due to the dispersion relation of LiNbO_3_ crystal (Fig. 4b).

## Discussion

It has been predicted that 3D NPCs can have unprecedented applications in nonlinear holography, nonlinear multiplexing, and multidimensional entanglement^40,41^. In this work, we have experimentally demonstrated its distinctive features in nonlinear multiplexing holography. The proposed QPM-division multiplexing method in 3D NPC provides a nonlinear holographic version of wavelength-division multiplexing (WDM). As the period number in 3D NPC is further increased to 200, the theoretical capacity of our method can reach 100 within a wavelength band of ~250 nm (see Supplementary Note 4), which is comparable to the performance of commercial WDM. In addition, the 3D QPM mechanism is fully utilized to substantially enhance the conversion efficiency of nonlinear holography. Besides, the QPM-division multiplexing can be realized by utilizing polarization, incident angle, and crystal temperature. Together with the enhanced security due to the reconstruction at a newly-generated wavelength, these unique characteristics distinguish our method from previous holographic schemes. QPM-division multiplexing nonlinear holography paves a way towards high-density and high-security storage of optical information.

## Materials and Methods

### Fabrication of 3D NPC

A regenerative amplified Ti:sapphire femtosecond laser system (Legend Elite-1K-HE, Coherent, USA) with 104 fs pulse width, 1 kHz repetition rate, and 800 nm central wavelength was employed to fabricate the 3D NPC in a commercial 5% MgO-doped LiNbO_3_ crystal. The laser power was modulated with a half-wave plate and a Glan laser beam splitter. After expansion, the laser beam was focused by an objective (50×, NA = 0.8, Olympus) into the LiNbO_3_ crystal which was mounted on a piezoelectric platform (E545, from Physik Instrumente GmbH & Co. KG, Germany) with nanometer resolution and 200 μm (*x*) × 200 μm (*y*) × 200 μm (*z*) moving ranges. The focal spot sizes inside the crystal are ~1.5 μm in *X* and *Y* directions and 3 μm in the *Z* direction. We could observe the fabricating process through a charge-coupled-device camera in real-time. The writing laser energies are 50, 60, 75, 95, 110, 125, 135, 145, 155, and 170 nJ from the top to bottom for ten layers along the *z*-axis with the corresponding exposed times of 70, 90, 110, 130, 150, 180, 220, 240, 260, and 280 ms, respectively. The fabrication volume was mainly limited by the performance of the used laser writing system.

### Experimental setup for characterizing QPM-division multiplexing nonlinear holography

The fundamental wave is generated by a Ti:sapphire femtosecond laser (Chameleon, Coherent) with a 75 fs pulse duration, 80 MHz pulse repetition rate, and a tunable wavelength ranging from 690 to 1050 nm. The power of the fundamental wave is controlled by a half-wave plate and a polarization beam splitter. The input beam is focused by a 100 mm lens and incident into the sample with its polarization along the *z*-axis to make use of the largest nonlinear coefficient *d*_33_ of LiNbO_3_ crystal. The beam waist at the focal point is about 40 μm, and the pump intensity is $1.9 \times 10^3{\mathop{\rm{W}}\nolimits} \cdot {\mathrm{mm}}^{ - 2}$. The output SH far-field patterns are projected onto a white screen and then recorded by a camera. A power meter is used to measure the SH powers.

## Supplementary information

Supplementary information

## Data Availability

The data that supports the results within this paper and other findings of the study are available from the corresponding authors upon reasonable request.

## Code availability

The custom code and mathematical algorithm used to obtain the results within this paper are available from the corresponding authors upon reasonable request.
