# Supplementary material for: Quasi-phase-matching-division multiplexing holography in a three-dimensional nonlinear photonic crystal
Source: Light Sci Appl. 2021 Jul 15;10:146. doi: 10.1038/s41377-021-00588-5 (PMC8282809; doi:10.1038/s41377-021-00588-5)
Supplement: Supplementary file 1 — Supplementary information [file 41377_2021_588_MOESM1_ESM.docx]

Supplementary information for

**Quasi-phase-matching-division multiplexing holography in a three-dimensional nonlinear photonic crystal**

Pengcheng Chen^1,4^, Chaowei Wang^2,4^, Dunzhao Wei^1,4^, Yanlei Hu^2,4^, Xiaoyi Xu^1^, Jiawen Li^2^, Dong Wu^2,*^, Jianan Ma^1^, Shengyun Ji^2^, Leran Zhang^2^, Liqun Xu^2^, Tianxin Wang^1^, Chuan Xu^1^, Jiaru Chu^2^, Shining Zhu^1^, Min Xiao^1,3,*^, and Yong Zhang^1,*^

^1^National Laboratory of Solid State Microstructures, College of Engineering and Applied Sciences, School of Physics, and Collaborative Innovation Center of Advanced Microstructures, Nanjing University, Nanjing 210093, China

^2^Hefei National Laboratory for Physical Sciences at the Microscale and CAS Key Laboratory of Mechanical Behavior and Design of Materials, Department of Precision Machinery and Precision Instrumentation, University of Science and Technology of China, Hefei 230026, China

^3^Department of Physics, University of Arkansas, Fayetteville, Arkansas 72701, USA

^4^These authors contribute equally to this work.

*To whom correspondence should be addressed: [zhangyong@nju.edu.cn](mailto:zhangyong@nju.edu.cn); dongwu@ustc.edu.cn; [mxiao@uark.edu](mailto:mxiao@uark.edu).

**This PDF file includes:**

Supplementary Notes 1 to 5

Supplementary Figures 1 to 9

Supplementary Table 1

**1. Comparison of nonlinear holography in 2D and 3D NPCs**

**
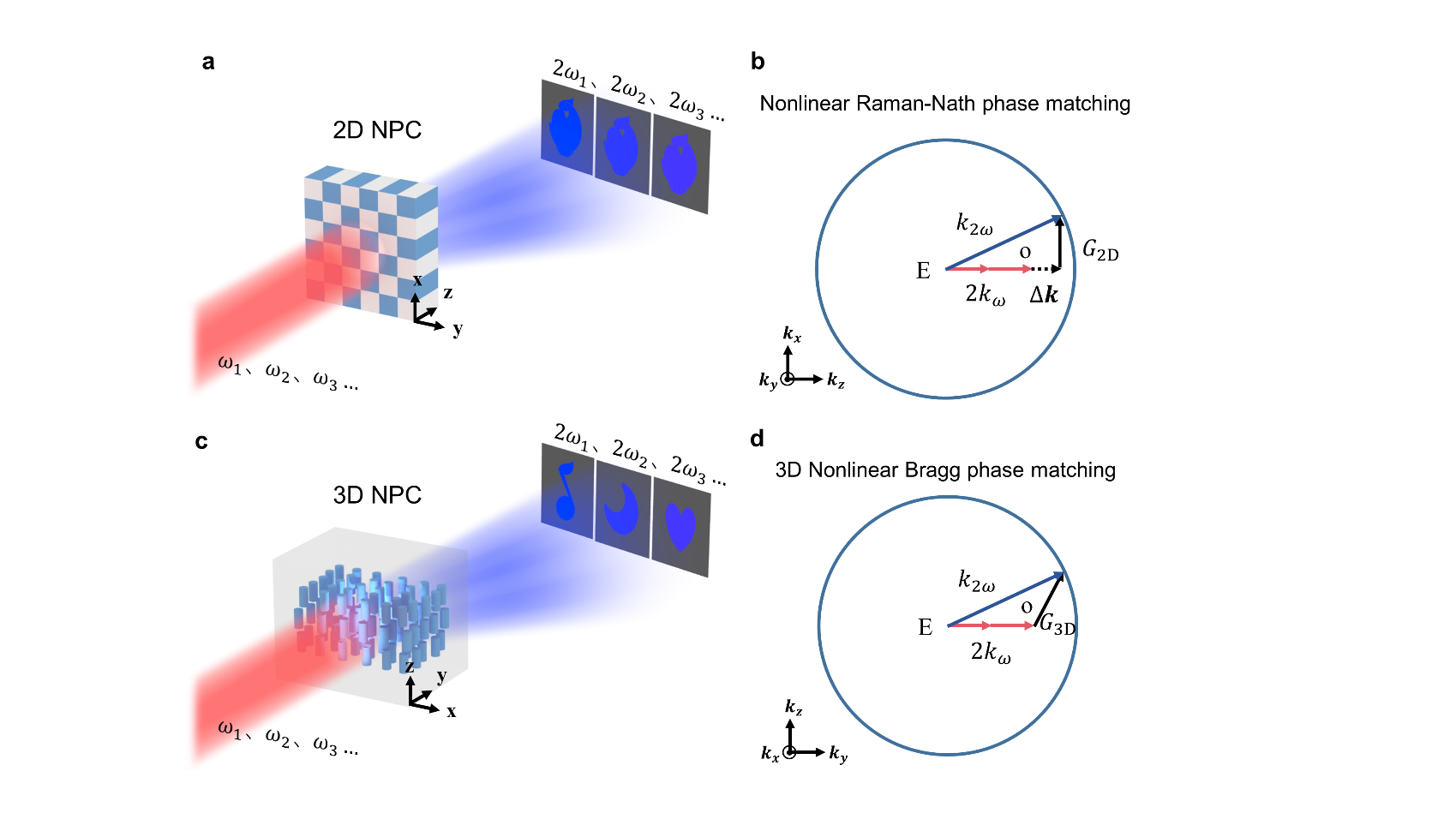
**

**Fig. S1** (a) The general configuration of nonlinear holography in 2D NPC. Multiple SH patterns of different optical frequencies cannot be well distinguished. (b) The diagram of nonlinear Raman-Nath phase matching in 2D NPC. (c) The scheme of nonlinear holography in 3D NPC, in which the SH patterns at different frequencies are well observed. (d) The complete QPM diagram in 3D NPC.

Fig. S1a shows the general configuration of nonlinear holography in 2D NPC. The fundamental wave is input along with *z* axis, and the SH wave is output under nonlinear Raman-Nath diffraction. Due to the lack of RVs along the propagation direction (Fig. S1b), there exists a phase mismatch that leads to poor conversion efficiency. In addition, the SH images encoded in various fundamental wavelengths cannot be well distinguished (Fig. S1a).

Fig. S1c shows the scheme for nonlinear holography in 3D NPC. The fundamental wave propagates along *y* axis so that one can use the maximal nonlinear coefficient *d*_33_ of LiNbO_3_ crystal. The 3D RVs are capable to fulfil the complete 3D QPM condition (Fig. S1d) for high conversion efficiency. Besides, multiple images can be distributed to different Ewald spheres in reciprocal space (see Fig. 1c in the manuscript). By altering the fundamental wavelength, one can tune the Ewald sphere, select various RV group to fulfil the corresponding QPM condition, and reconstruct SH images separately. In our experiment, the wavelength interval between neighboring channels is larger than the wavelength bandwidth of the used femtosecond laser. Therefore, the crosstalk between different channels can be ignored. For practical applications, one can use a narrow-linewidth nanosecond laser to fully utilize the capacity of 3D NPC.

**2. 3D Iterative Fourier Transform algorithm**

In our method, we use a modified 3D iterative Fourier transform algorithm to transform the ideal RV distribution *F*_0_ in reciprocal space to a 3D phase hologram *H* in real space. According to our fabrication capability, the pixel number of NPC model in our calculation is set to be 16 (*N_x_*) × 16 (*N_y_*) × 10 (*N_z_*). To match the point-by-point laser writing, we use the detour phase to design the phase hologram in our algorithm, in which the phase of each pixel is determined by the relative position of the laser-erased area in a cuboid unit. The schematic flow of the algorithm is shown in Fig. 2a of the manuscript. According to the target images at their respective wavelengths, multiple RV groups are calculated through 3D QPM condition, which form an ideal RV distribution (*F*_0_). *F*_0_ is set as the initial input in our algorithm. One complete loop includes 3 steps. First, 3D inverse Fourier transform of *F*_0_ produces a 3D complex hologram, from which the phase term is selected to compose a 3D phase hologram *H*_0_. Second, Fourier transform of *H*_0_ gives its corresponding RV distribution ($F_{0}^{'}$). Third, we apply the following constraint condition to $F_{0}^{'}$,

$F_{q+1}=\left\{ \begin{aligned} \left( c_{1}\left| F_{0} \right|+c_{2}\left| F_{q}^{'} \right| \right)\times\frac{F_{0}}{\left| F_{0} \right|} \vec{G}\in\left[ \vec{G}_{E}-\Delta G,\vec{G}_{E}+\Delta G \right] \\ c_{3}F_{q}^{'} \vec{G}\notin\left[ \vec{G}_{E}-\Delta G,\vec{G}_{E}+\Delta G \right] \end{aligned} \right.$ (S1)

with *q* = 0, 1, 2, …… *Q*. Here, $c_{1}$, $c_{2}$, and $c_{3}$ are related to the fidelity of the reconstruction, i.e., the similarity between the reconstructive image and the target one. $\vec{G}_{E}$ represents the RVs located at the designated Ewald sphere. $\left[ \vec{G}_{E}-\Delta G,\vec{G}_{E}+\Delta G \right]$ defines the range of the reciprocal space, where RVs are precisely optimized to approach to those on *F*_0_. The RV band out of this range is defined as the saved band, which can be used to further enhance the image quality. Fig. S2 shows the effect of the saved RV band, in which one can see a clear improvement in the designated RV space. The quality of the reconstructive image strongly depends on the fabrication capability, such as voxel number, structure uniformity (especially along the depth direction), and so on. The saved RV band can partially compensate for the limitations in fabrication.


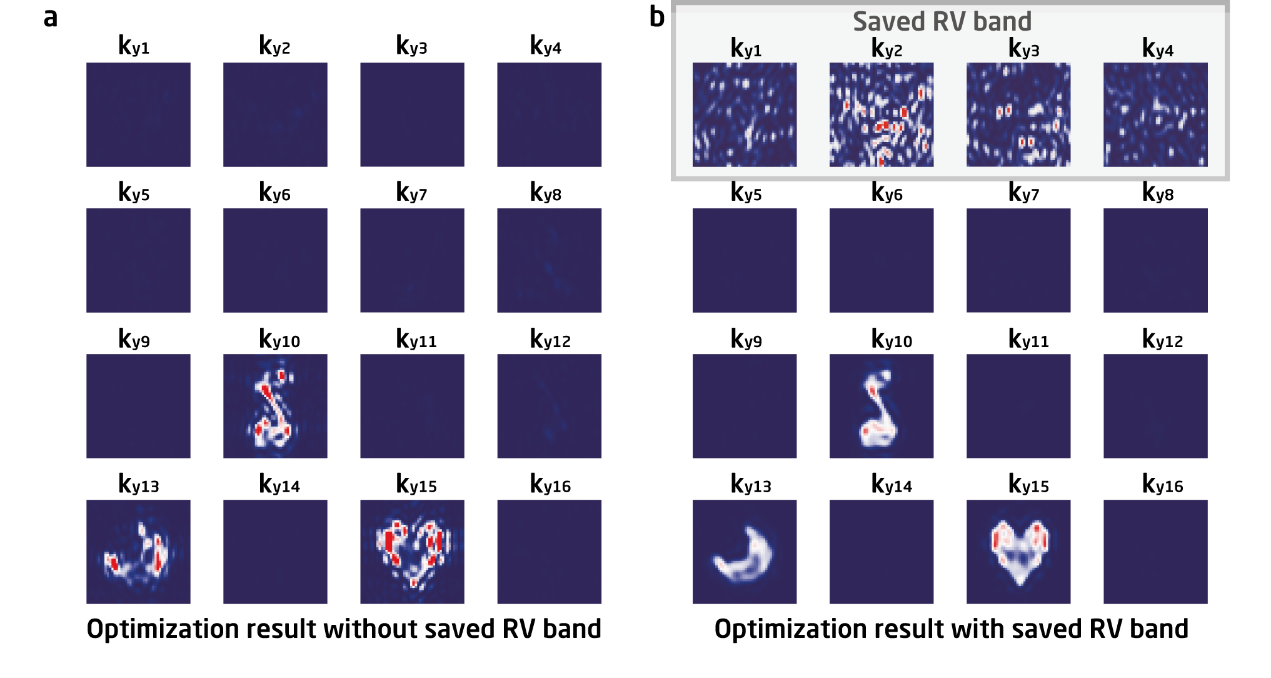
**Fig. S2** The simulated RV distributions without (a) and with (b) using the saved RV band.

After performing Eq. (S1), we produce the next-generation RV distribution *F*_1_ and finish one complete loop. Then, we repeat this loop by *Q* iterations. When *Q* is big enough, we can get an optimized $F_{Q}^{'}$ and *H_Q_*. The calculated 3D phase holograms are shown in Fig. S3.


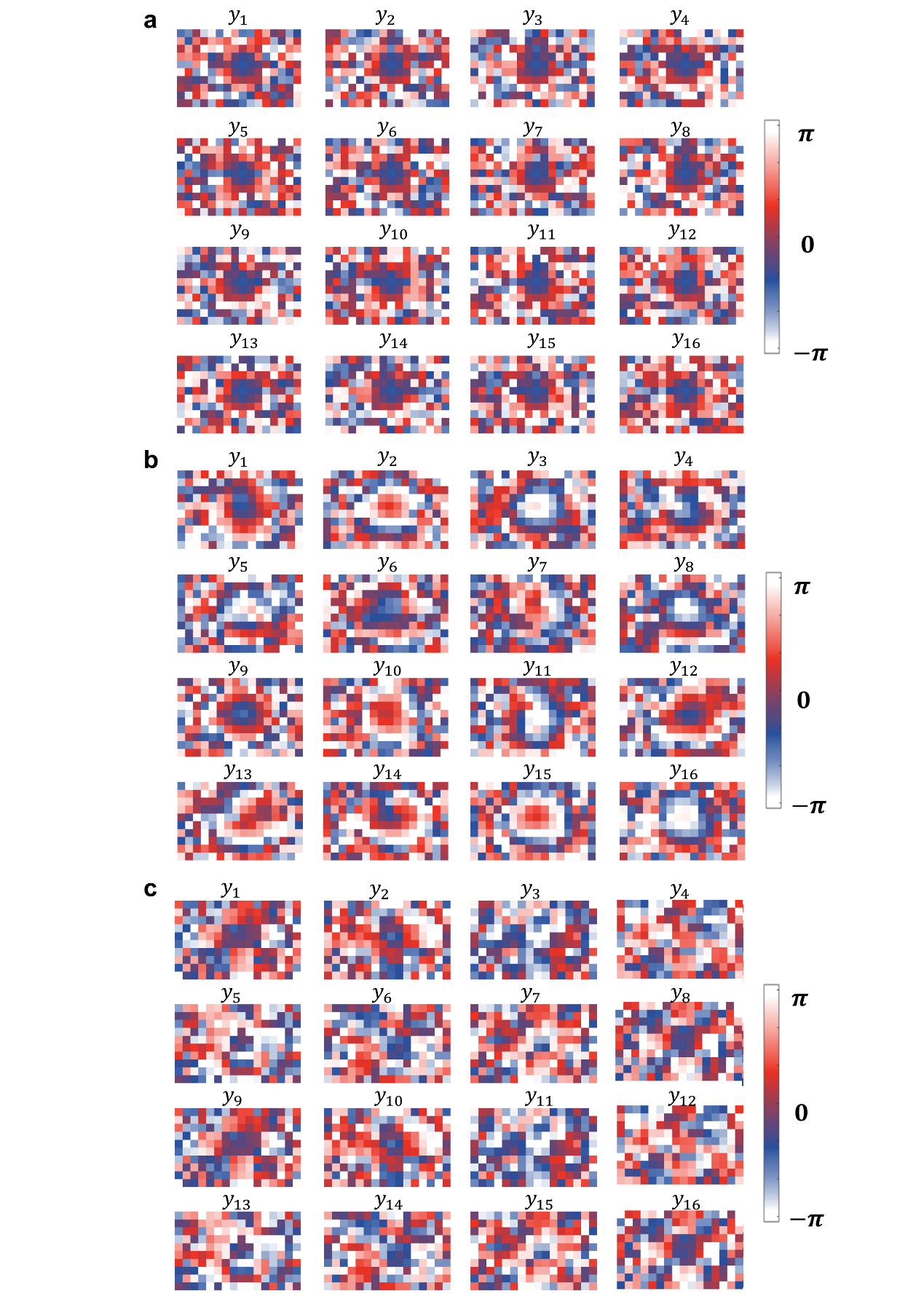


**Fig. S3** The calculated 3D phase holograms (a-c) correspond to Figs. 3a, 3c, and 4b in the manuscript, respectively. To match the femtosecond-laser erasing technique, 3D phase hologram consists of 16 (*x*) × 16 (*y*) × 10 (*z*) pixels. *y*_1_ - *y*_16_ represent the 16 layers along y direction.

**3. 3D detour phase coding**

In our experiment, the 3D NPC is fabricated by femtosecond laser erasing technique. Therefore, the 3D phase hologram is converted to a binary amplitude-modulated *χ*^(2)^ structure through detour phase coding.

The fabrication region is divide into *M* (*x*) × *N* (*y*) × *J* (*z*) unit cells. Each unit is a cuboid of *w* (*x*) × *l* (*y*) × *h* (*z*) (Fig. 2c in the manuscript). Inside each cuboid unit, we use femtosecond laser to fabricate a cylinder with its *χ*^(2)^ being erased. The height and radius of the cylinder are *h*_c_ and *r*_c_, respectively. The detour phase is realized by tuning the relative position *α* of the cylinder position along *x*-axis.

Consider that 3D phase hologram *H* has a RV distribution *F*_0_, satisfying $\mathcal{F}\left( e^{iH} \right)=F_{0}$. Here, $\mathcal{F}$ represents 3D Fourier transform. The structure of 3D NPC after detour phase coding is written as $f=1-\left( 1-\nu\right)\times S$. Here, $\nu$ is modulation depth of *χ*^(2)^ and *S* is defined by

$S=\sum_{mnj} \delta\left( x-mw-\alpha_{mnj},y-nl,z-jh \right)\otimes\left[ rect\left( \frac{z}{h_{c}} \right)circ\left( \frac{\sqrt{x^{2}+y^{2}}}{r_{c}} \right) \right]$ (S2)

where *m*, *n*, and *j* are positive integers. $\alpha_{mnj}$ represents the displacement of each laser-erased cylinder. After Fourier transform of Eq. (S2), one can obtain the corresponding RV distribution^1^

$F=\pi r_{c}^{2}\frac{2J_{1}\left( r_{c}\sqrt{k_{x}^{2}+k_{y}^{2}} \right)}{r_{c}\sqrt{k_{x}^{2}+k_{y}^{2}}}\times h_{c}sinc\left( k_{z}h_{c} \right)\times\sum_{mnj} e^{ik_{x}\alpha_{mnj}}e^{i\left( k_{x}mw+k_{y}nl+k_{z}jh \right)}$ (S3)

Here, *k_x_*, *k_y_*, and *k_z_* are the coordinates in reciprocal space. Considering the first two terms in the right side of Eq. (S3) are slowly-varying, one can obtain a RV distribution close to *F*_0_ when $k_{x}\alpha_{mnj}=H_{mnj}$. Here, *H_mnj_* represents the phase of each unit in 3D phase hologram. Therefore, $\alpha_{mnj}=\frac{wH_{mnj}}{2I\pi}$ with *I* = 2 in our experiment to avoid the disturbance from neighboring structures. Considering the fabrication precision, the detour phase ($0-2\pi$) is discretized to 30 levels and then is transformed to the relative position of $\alpha_{mnj}$. The calculated 3D NPC structures are shown in Fig. S4 and Table S1.


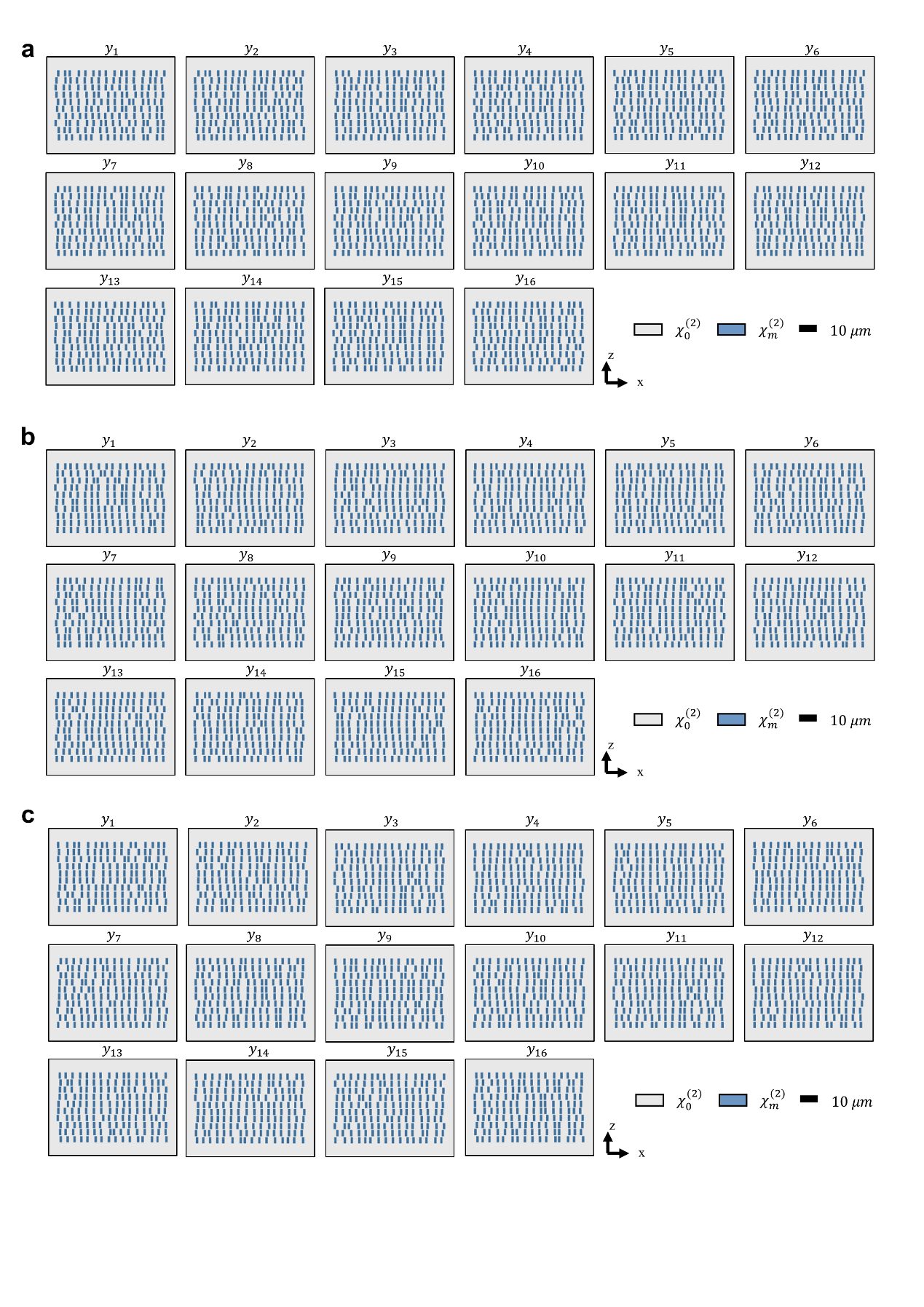


**Fig. S4** Based on detour phase coding, we calculate the 3D NPC structures (a-c) that correspond to Figs. 3a, 3c, and 4b in the manuscript, respectively. Note that the phase of 3D hologram is divided into 30 levels. Each NPC structure has 16 (*x*) × 16 (*y*) × 10 (*z*) pixels. *y*_1_ - *y*_16_ represent the 16 layers along y direction.

**Table S1** We use the *y*_1_ layer in Fig. S4a as an example. *α_mnj_* (μm) is the displacement along x axis in each pixel and *H_mnj_* (rad) represents the corresponding phase. The subscripts *m*, *n*, and *j* are the pixel indices along *x*, *y*, and *z* axis, respectively.

| ***m*** | **1** | **2** | **3** | **4** | **5** | **6** | **7** | **8** | **9** | **10** | **11** | **12** | **13** | **14** | **15** | **16** |
| --- | --- | --- | --- | --- | --- | --- | --- | --- | --- | --- | --- | --- | --- | --- | --- | --- |
| ***H_m,1,1_*** | 4.74 | 4.41 | 0.01 | 3.75 | 2.69 | 4.62 | 2.78 | 1.82 | 4.68 | 4.56 | 0.84 | 1.93 | 5.83 | 6.20 | 4.16 | 5.17 |
| ***α_m,1,1_*** | 1.52 | 1.38 | 0.00 | 1.17 | 0.83 | 1.45 | 0.90 | 0.55 | 1.52 | 1.45 | 0.28 | 0.62 | 1.86 | 2.00 | 1.31 | 1.66 |
| ***H_m,1,2_*** | 1.82 | 4.99 | 1.48 | 0.49 | 1.94 | 1.65 | 1.19 | 2.63 | 2.74 | 1.42 | 0.85 | 1.01 | 4.04 | 5.47 | 2.11 | 0.46 |
| ***α_m,1,2_*** | 0.55 | 1.59 | 0.48 | 0.14 | 0.62 | 0.55 | 0.41 | 0.83 | 0.90 | 0.48 | 0.28 | 0.34 | 1.31 | 1.72 | 0.69 | 0.14 |
| ***H_m,1,3_*** | 0.38 | 1.93 | 0.31 | 2.80 | 2.41 | 3.13 | 1.56 | 1.50 | 0.19 | 1.14 | 0.42 | 2.86 | 3.75 | 2.82 | 1.54 | 1.81 |
| ***α_m,1,3_*** | 0.14 | 0.62 | 0.07 | 0.90 | 0.76 | 0.97 | 0.48 | 0.48 | 0.07 | 0.34 | 0.14 | 0.90 | 1.17 | 0.90 | 0.48 | 0.55 |
| ***H_m,1,4_*** | 1.58 | 1.72 | 1.23 | 1.53 | 5.08 | 5.93 | 1.38 | 5.78 | 5.31 | 5.46 | 1.36 | 4.64 | 3.15 | 1.08 | 2.44 | 2.87 |
| ***α_m,1,4_*** | 0.48 | 0.55 | 0.41 | 0.48 | 1.59 | 1.86 | 0.41 | 1.86 | 1.72 | 1.72 | 0.41 | 1.45 | 1.03 | 0.34 | 0.76 | 0.90 |
| ***H_m,1,5_*** | 4.23 | 4.16 | 1.73 | 3.47 | 5.81 | 5.91 | 6.23 | 5.36 | 5.03 | 5.34 | 5.60 | 0.14 | 5.16 | 2.18 | 0.79 | 0.06 |
| ***α_m,1,5_*** | 1.38 | 1.31 | 0.55 | 1.10 | 1.86 | 1.86 | 2.00 | 1.72 | 1.59 | 1.72 | 1.79 | 0.07 | 1.66 | 0.69 | 0.28 | 0.00 |
| ***H_m,1,6_*** | 2.70 | 0.86 | 4.19 | 3.14 | 4.39 | 1.60 | 0.95 | 5.74 | 5.36 | 5.71 | 0.07 | 2.97 | 1.94 | 4.35 | 5.30 | 1.27 |
| ***α_m,1,6_*** | 0.83 | 0.28 | 1.31 | 0.97 | 1.38 | 0.48 | 0.28 | 1.86 | 1.72 | 1.79 | 0.00 | 0.97 | 0.62 | 1.38 | 1.66 | 0.41 |
| ***H_m,1,7_*** | 6.09 | 5.85 | 5.39 | 5.78 | 3.98 | 3.13 | 1.72 | 0.35 | 0.11 | 0.34 | 0.19 | 2.16 | 5.07 | 4.27 | 4.39 | 0.75 |
| ***α_m,1,7_*** | 1.93 | 1.86 | 1.72 | 1.86 | 1.24 | 0.97 | 0.55 | 0.14 | 0.07 | 0.14 | 0.07 | 0.69 | 1.59 | 1.38 | 1.38 | 0.21 |
| ***H_m,1,8_*** | 0.23 | 6.09 | 0.44 | 1.77 | 5.95 | 4.71 | 5.54 | 1.76 | 2.00 | 1.36 | 5.59 | 4.10 | 5.98 | 0.66 | 5.84 | 3.47 |
| ***α_m,1,8_*** | 0.07 | 1.93 | 0.14 | 0.55 | 1.86 | 1.52 | 1.79 | 0.55 | 0.62 | 0.41 | 1.79 | 1.31 | 1.93 | 0.21 | 1.86 | 1.10 |
| ***H_m,1,9_*** | 5.68 | 3.61 | 0.72 | 2.03 | 2.82 | 3.16 | 4.13 | 3.33 | 4.43 | 5.26 | 0.85 | 0.34 | 4.84 | 5.64 | 6.27 | 2.59 |
| ***α_m,1,9_*** | 1.79 | 1.17 | 0.21 | 0.62 | 0.90 | 1.03 | 1.31 | 1.03 | 1.38 | 1.66 | 0.28 | 0.14 | 1.52 | 1.79 | 2.00 | 0.83 |
| ***H_m,1,10_*** | 6.12 | 6.25 | 4.11 | 5.97 | 4.01 | 0.92 | 5.37 | 5.33 | 4.75 | 3.98 | 3.73 | 0.91 | 6.06 | 0.79 | 4.81 | 2.50 |
| ***α_m,1,10_*** | 1.93 | 2.00 | 1.31 | 1.93 | 1.24 | 0.28 | 1.72 | 1.72 | 1.52 | 1.24 | 1.17 | 0.28 | 1.93 | 0.28 | 1.52 | 0.83 |

**4. Capacity in QPM-division multiplexing holography**


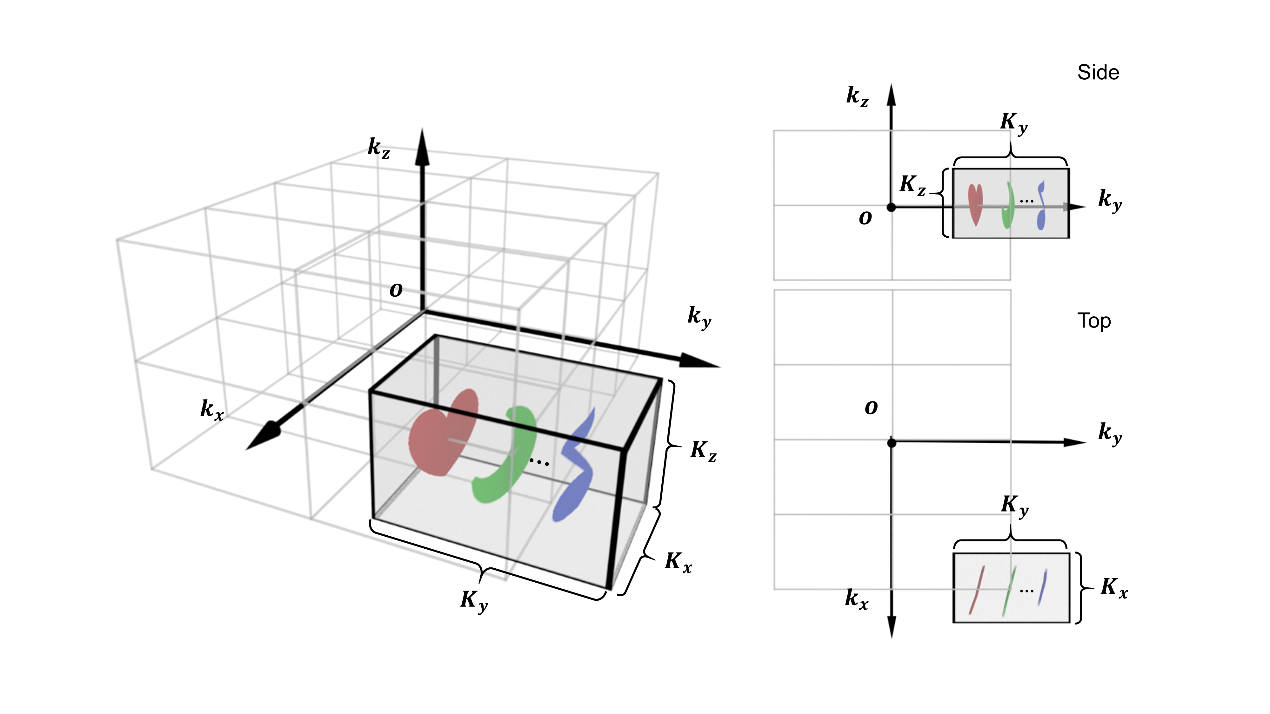


**Fig. S5** The RV distribution in reciprocal space. Because of the unit size in 3D NPC, the RV source for nonlinear holography is within a box of ${Band}_{G}=\frac{2\pi}{w}\left( k_{x} \right)\times\frac{2\pi}{l}\left( k_{y} \right)\times\frac{2\pi}{h}\left( k_{z} \right)$.

We analyze the multiplexing capacity in reciprocal space. The 3D NPC has *M* (*x*) × *N* (*y*) × *J* (*z*) units. The unit cell has a size of *w* (*x*) × *l* (*y*) × *h* (*z*) as shown in Fig. 2c of the manuscript. The total RV bandwidth that available for nonlinear holography is ${Band}_{G}=\frac{2\pi}{w}\left( k_{x} \right)\times\frac{2\pi}{l}\left( k_{y} \right)\times\frac{2\pi}{h}\left( k_{z} \right)$ (i.e., the box area in Fig. S5). Because the SH wave vector is far larger than RVs in the box, the cross section between Ewald sphere and the RV box can be approximately seen as a plane. In this case, the components in *x-z* plane ($\frac{2\pi}{w}\left( k_{x} \right)\times\frac{2\pi}{h}\left( k_{z} \right)$) decides the area of the reconstructive SH images. The available RV bandwidth along propagation direction is $\frac{2\pi}{l}\left( k_{y} \right)$, which can be distributed to the SH images at different QPM wavelengths.

The intensity of the generated SH pattern is proportional to $\mathrm{sinc}^{2}\left( \frac{\Delta kNl}{2} \right)$, which decides the profile of the QPM peaks. Here, $\Delta k=k_{2\omega,y}-2k_{\omega,y}-G_{y}$ is the phase mismatch along *k*_y_ axis with *G_y_* being the *y* component of RV. To effectively avoid the crosstalk between different channels, we define the limit as that two neighboring QPM peaks overlap at their first zero-value points (i.e.,$\mathrm{sinc}^{2}\left( \frac{\Delta kNl}{2} \right)=0$), which requires $\Delta k=\frac{2\pi}{Nl}$ and $-\frac{2\pi}{Nl}$ for two neighboring QPM peaks, respectively. So, the required RV bandwidth for each SH image is $\Delta G=\frac{4\pi}{Nl}$. The maximal channel number is $\frac{{Band}_{G}\left( k_{y} \right)}{\Delta G}=\frac{N}{2}$ in theory. In our scheme, the required RV bandwidth is the same for each image. Correspondingly, the required wavelength bandwidth increases along with the fundamental wavelength because of the dispersion relation of LiNbO_3_ crystal.

In our experiment, the unit size is 4 *μm* (*x*) × 3 *μm* (*y*) × 4 *μm* (*z*) and the period number is 16 along *y* direction. The available RV bandwidth along propagation direction is $\frac{2\pi}{3} {\mu m}^{-1}$, the required RV bandwidth for each image is $\frac{\pi}{12} {\mu m}^{-1}$, and the theoretical multiplexing capacity is 8 images. In experiment, the situation is more complicated. Normally, the SH power in each image decreases when the channel number increases (if the period number is not changed). In our experiment, the period number is limited and we save proper RV band to achieve high-quality image reconstruction. As a result, the experimental multiplexing capacity is a little less than the theoretical value. For instance, the multiplexing capacity in our experiment is 6 images considering the image quality.


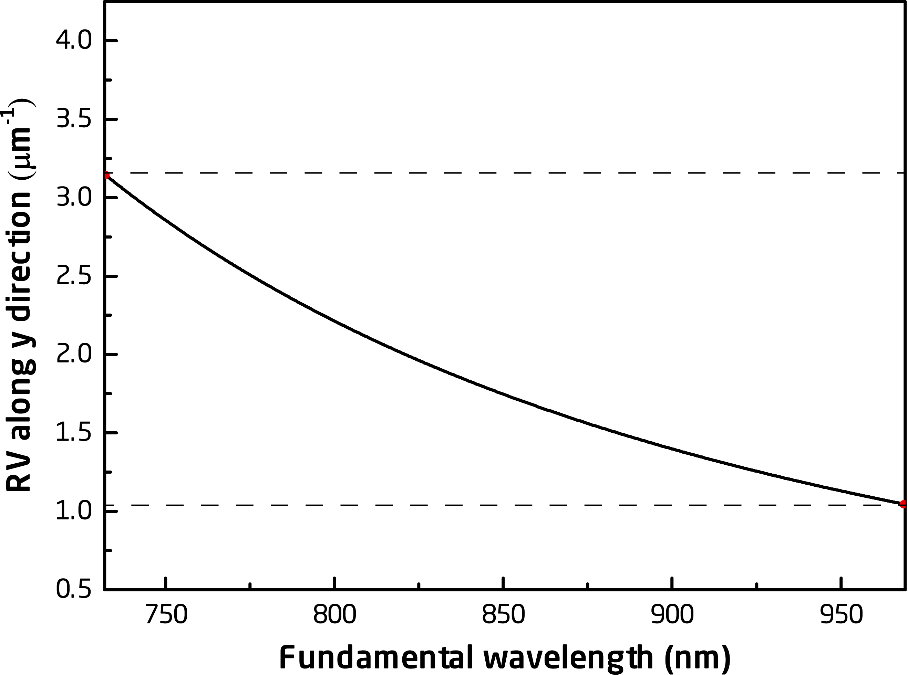


**Fig. S6** The available RVs along *y* direction and the corresponding QPM wavelengths.

The multiplexing capacity can be further enhanced by fabricating a larger 3D NPC. Assume that the sample is 600 *μm* long in *y* direction (i.e., 200 periods). The required RV bandwidth for each image is reduced to $\frac{\pi}{150} {\mu m}^{-1}$. The available RV along the propagation direction *y* is from $\frac{\pi}{3} {\mu m}^{-1}$ to $\pi{\mu m}^{-1}$, and the corresponding QPM wavelength varies from 732 nm to 969 nm (Fig. S6). In this case, the theoretical multiplexing capacity can reach 100 within a wavelength band of 237 nm. In our algorithm, the required RV bandwidth is equal for each channel. Because of the dispersion of LiNbO_3_ crystal, the wavelength interval of the neighboring channels ranges from 1.2 nm to 4.8 nm (Fig. S7).





**Fig. S7** The fundamental wavelength interval between the adjacent channels.

Next, we calculate the pump power for 100-channel nonlinear holography. In the non-depletion approximation, the average SH power in each image satisfies $\bar{P}_{SH}\propto\frac{N_{y}^{2}P_{FW}^{2}}{N}$, where *N_y_* is the period number along *y*-axis, *N* is the channel number, and *P*_FW_ is the pump power. To obtain the same average SH power as in this work, *P*_FW_ should satisfy $\frac{{16}^{2}\left( 2.4 W \right)^{2}}{6}=\frac{N_{y}^{2}\left( P_{FW} \right)^{2}}{N}$. When *N_y_* = 200 and *N* = 100, the pump power is calculated to be 783.8 mW. If we increase *N_y_* to 500, the pump power can be reduced to 313.5 mW. Fig. S8 shows an example to simulate 100-channel nonlinear holography with *N_y_* = 267. Here, 25% of the total RV band is saved to enhance the image quality.


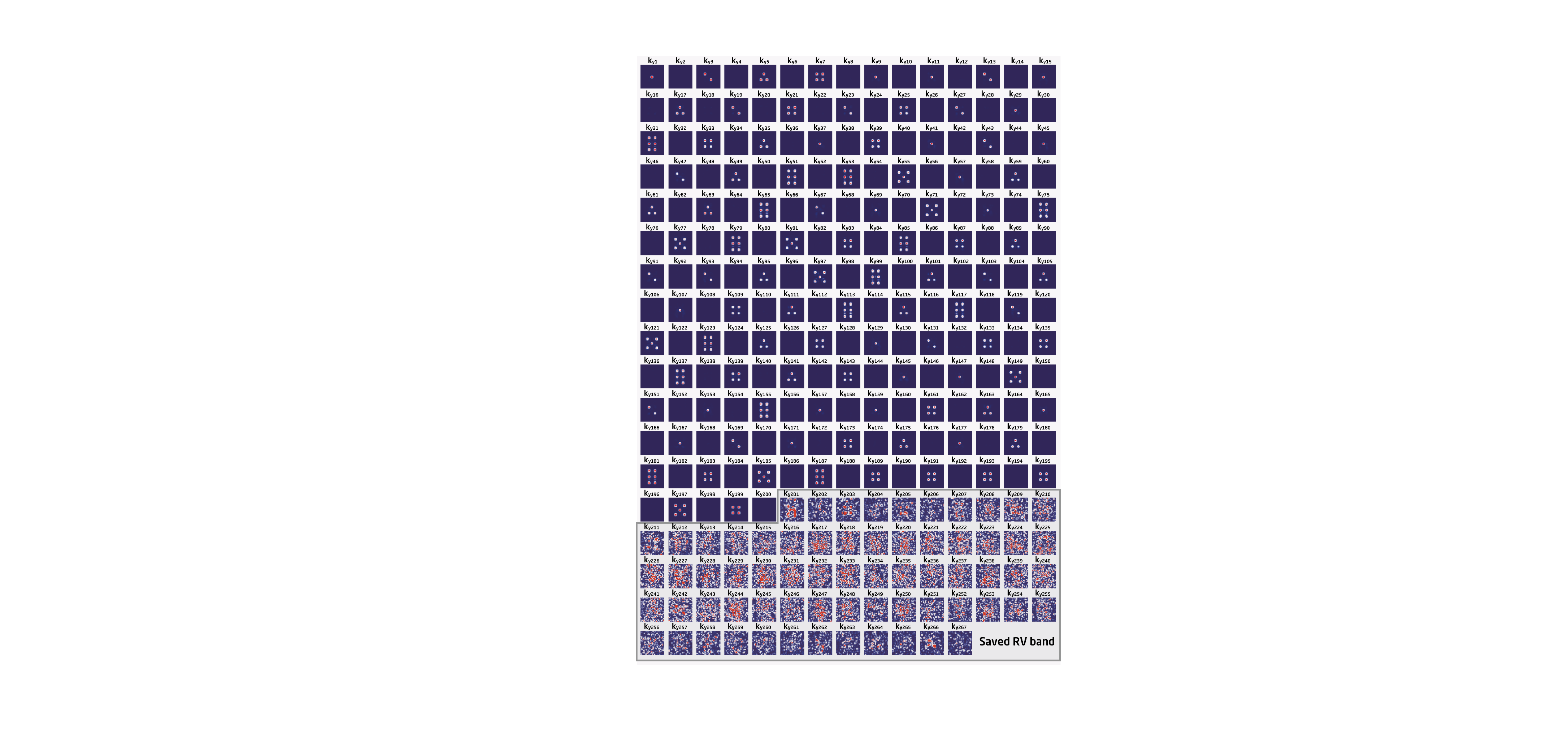


**Fig. S8** The simulation of 100-channel nonlinear holography. Here, about 25% of the total RV band is saved to enhance the image quality.

**5. The loss of 3D NPC**

The main loss of 3D NPC are the scattering and diffraction caused by the slight linear refractive index change during laser writing process. These losses are measured to be about 1.8%. The linear diffraction pattern of 3D NPC is shown in Fig. S9a, in which the ±1-order diffraction spots are far weaker than the central spot. We also record the pattern by passing the fundamental beam through a uniform crystal for comparison (Fig. S9b). One can see no significant differences in the central spots.


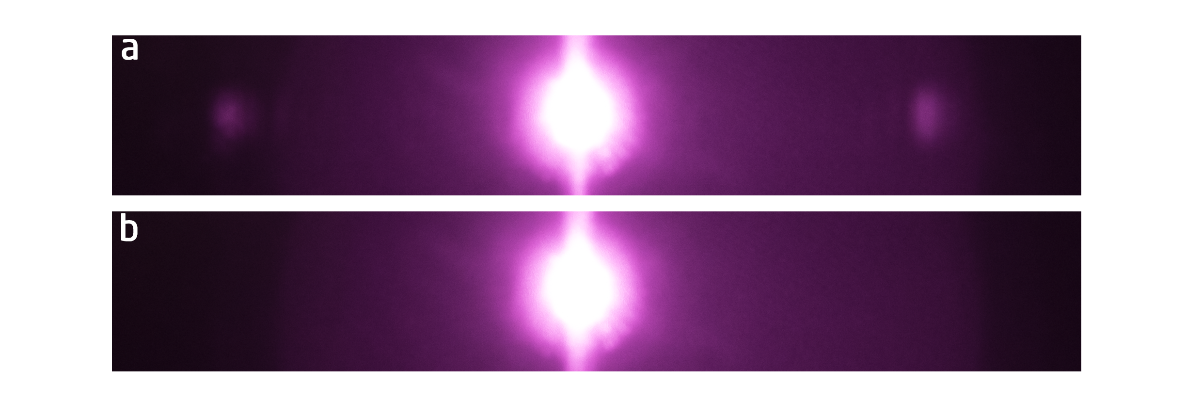


**Fig. S9** By using a fundamental beam of 811 nm, we record the diffraction pattern of the 3D NPC (a) and the pattern through a uniform crystal for comparison (b).

**References**

(1). Goodman, J.W. Introduction to Fourier optics. (McGraw-Hill, New York, 1968).
